# Long-Term Prospective Study of 6104 Survivors of Arsenic Poisoning During Infancy Due to Contaminated Milk Powder in 1955

**DOI:** 10.2188/jea.JE20090131

**Published:** 2010-11-05

**Authors:** Hideo Tanaka, Hideaki Tsukuma, Akira Oshima

**Affiliations:** 1 Division of Epidemiology and Prevention, Aichi Cancer Center Research Institute; 2 Department of Cancer Control and Statistics, Osaka Medical Center for Cancer and Cardiovascular Diseases; 3 Cancer Information Services, Osaka Medical Center for Cancer and Cardiovascular Diseases

**Keywords:** arsenic poisoning, mortality, prospective study, food poisoning, standardized mortality ratio

## Abstract

**Background:**

In 1955, an outbreak of arsenic poisoning caused by ingestion of arsenic-contaminated dry milk occurred in western Japan. We assessed the excess mortality among Japanese who were poisoned during this episode as infants.

**Methods:**

We identified and enrolled 6104 survivors (mean age at enrollment, 27.4 years) who had ingested contaminated milk when they were age 2 years or younger; they were followed until 2006 (mean duration of follow-up, 24.3 years). Death certificates of subjects who died between 1982 and 2006 were examined to calculate cause-specific standardized mortality ratios (SMRs) using the mortality rate among Osaka residents as the standard.

**Results:**

There was no significant excess overall mortality (SMR: 1.1, 95% confidence interval: 1.0–1.2). However, significant excess mortality in both sexes was observed from diseases of the nervous system (3.7, 1.9–6.2). Excess mortality from all causes of death decreased to unity beyond 10 years after study enrollment. The 408 men who were unemployed at the time of enrollment in the study had a significantly elevated risk of death from diseases of the nervous system (25.3, 10.8–58.8), respiratory diseases (8.6, 3.1–16.8), circulatory diseases (3.2, 1.6–5.2), and external causes (2.6, 1.4–4.1).

**Conclusions:**

As compared with the general population, survivors of arsenic poisoning during infancy had a significantly higher mortality risk from diseases of the nervous system.

## INTRODUCTION

Arsenic toxicity due to ingestion of arsenic-contaminated drinking water from natural geological sources and local exposure to anthropogenic emissions from mining, smelting, and agricultural sources (pesticides or fertilizers) is a global health issue.^1^ Several ecological, prospective, and case-control studies have assessed the effects of chronic arsenic exposure due to drinking water and have showed that arsenic is carcinogenic to skin, lung, bladder, kidney, and liver.^2^^–^^12^ Prospective studies showed that chronic arsenic exposure is also associated with hyperkeratosis, ischemic heart disease,^13^ cerebrovascular disease,^14^ type 2 diabetes,^15^ and peripheral neuropathy.^16^ However, most of these studies investigated adults at the time of arsenic exposure.

In the early summer of 1955, an outbreak of arsenic poisoning from ingestion of arsenic-contaminated milk powder occurred in western Japan.^17^^,^^18^ Arsenic contamination happened during the production of Morinaga Dry Milk at the Tokushima plant on Shikoku Island. The source of arsenic contamination was a disodium phosphate product added to cow’s milk as a stabilizer to maintain constant acidity. This particular industrial-grade disodium phosphate product was of low purity and contained 5% to 8% arsenic (expressed as arsenious acid).^19^^,^^20^ Records at Morinaga showed that approximately 460 000 one-pound cans of arsenic-contaminated dry milk were produced between April and August of 1955. The National Hygiene Institute and a collaborating university analyzed samples from 76 cans and found that the median concentration of arsenious acid in the dry milk samples was 30 mg/kg (range, 1 to 60 mg/kg). The Morinaga Milk Company began to recall cans of the arsenic-contaminated dry milk under the direction of the Ministry of Health and Welfare of Japan on 24 August 1955, and the poisoning outbreak had stopped by the end of August 1955. However, 12 131 infants had symptoms of arsenic poisoning due to drinking the dry milk substitute, and 130 died by June 1956.^18^ The patients had fever, skin pigmentation, hepatomegaly, and anemia, which are known clinical symptoms of arsenic poisoning. Based on the average volume of milk consumed, it was estimated that the daily arsenic intake would have been about 2.5 mg for a 1-month-old infant, 3.2 mg for a 2-month-old infant, and 4.6 mg for a 6-month-old infant.^19^ Although it is likely that many children who drank the dry milk developed chronic symptoms of nervous system disorders due to the arsenic poisoning, there was no systematic social support or financial aid from the Morinaga Milk Company until 1973. This serious food poisoning accident and its health effects have not been documented in the English literature, although Dakeishi et al^21^ recently published a review of several studies of the victims.

In 1974, the Hikari Kyokai Foundation was established to aid persons who drank the arsenic-contaminated Morinaga Dry Milk and had any symptom of arsenic poisoning during infancy. The Foundation, which is financially supported by the Morinaga Milk Company, provides health check-ups, health counseling, and financial support for the health and welfare of the victims. In 1982, the Foundation sponsored a prospective cohort study to investigate the long-term adverse effects of arsenic exposure due to ingestion of Morinaga Dry Milk during infancy. Several epidemiologists in Osaka, including the present authors, began studying the exposed individuals when the survivors were 27 years of age.^22^ The current analysis assessed excess mortality and causes of death from 1982 through 2006 among survivors who had ingested arsenic-contaminated milk powder during infancy.

## METHODS

In April 1982, the Foundation aided 8114 survivors who ingested arsenic-contaminated milk in 1955. Of these, 6394 (78.8%) gave consent to Foundation staff to be followed as part of the study. For the present analysis, we selected 6227 individuals (97.4%) who were younger than 2 years at the time of the outbreak. Follow-up was started in May 1982, and the individuals were contacted by telephone, home visit, and/or post several times a year by non-medical Foundation staff. Of these 6227 individuals, we excluded 123 (2.0%) who were lost to follow-up by the end of 2006. The remaining 6104 cases were included in the current analysis. Approximately 70% of the subjects lived in the Kinki, Chugoku, or Shikoku regions at the time of enrollment.

When the staff were informed of the death of a subject, the Foundation asked his or her family to submit a copy of the death certificate for payment of condolence money. The date of death was obtained from the death certificate, as was the underlying cause of death, which was classified by one of the authors according to the International Classification of Diseases, 9th Revision.

We calculated the number of person-years at risk from the date of entry into the study (1 May 1982) to the end of follow-up (date of death or 31 December 2006, whichever was earlier) for each subject and classified them by sex, 5-year age band, and 5-year calendar period. We then compared the observed number of cause-specific deaths with the expected number of deaths. The expected number of deaths was obtained by multiplying the number of person-years at risk—stratified by sex, 5-year age band, and 5-year calendar period—with cause-specific mortality rates among Osaka residents in each corresponding stratum, which were estimated in the National Vital Statistics Data. We used the mortality rate in Osaka prefecture (population size 8.7 million) as the reference rate because most study subjects lived in the Kansai area, ie, western Japan, which includes Osaka prefecture, the most populous prefecture in western Japan.

We calculated the standardized mortality ratio (SMR) as the ratio of observed deaths to the expected number of deaths. In the analysis of mortality, we considered the following causes of death: infectious diseases (ICD-9; 001.0–139.8), malignant diseases (140.0–205.9, 230.0–234.9); endocrine, metabolic, and immune disorders (240.0–279.9); psychiatric diseases (290.0–319); diseases of the nervous system (320.0–389.9); diseases of the circulatory system (390.0–459.9); diseases of the respiratory system (460.0–519.9); diseases of the digestive organs (520.0–579.9); diseases of skeletal muscle and connective tissue (710.0–739.9); congenital anomalies (740.0–759.9); and external causes (E800–E999). Unfortunately, we had no information on the level of arsenic exposure from ingestion of Morinaga Dry Milk or the severity of arsenic poisoning in any individual. Therefore, as an indicator of the severity of arsenic poisoning, we classified the study subjects into 2 subgroups according to whether they were employed or not at the time the study started (April 1982). This information was obtained from a survey to assess exposed individuals’ need for aid, which was conducted by the Foundation in 1982 through 1984.

Statistical tests on the SMR were performed based on the assumption that the observed number of deaths followed a Poisson distribution. If the 95% confidence interval (95% CI) did not include 1.00, the SMR was considered to be statistically significant (*P* < 0.05). Data analyses were performed with the SAS/PC statistical package (SAS Institute, Cary, NC).

Of the 6104 study subjects, 1097 (18.0%) who had initially consented to participate in this study subsequently declined to be actively contacted by the Foundation by the end of 2006. We obtained information on 11 deaths among these 1097 individuals through notification by their families for the purpose of receiving condolence money. The remaining 1086 individuals were assumed to be alive until the end of 2006 because their families did not request condolence money during the follow-up period. Because the 1097 individuals were thought to be relatively healthy and to have less need for aid by the Foundation, this may have led to overestimation of mortality. Therefore, we performed additional SMR calculations in which the 1097 individuals were excluded (Appendix Tables A1–A3).

This study protocol was approved by the Ministry of Health and Welfare of Japan. The authors received permission to submit the current manuscript to this journal from the Hikari Kyokai Foundation in July 2008. We did not submit the study protocol to the ethics committees of our institute because the study was started before the Japanese ethical guidelines for epidemiological research were issued in 2002, and because the Ministry of Health and Welfare had already approved the study protocol at the beginning of the study in 1982.

## RESULTS

The mean age of the 6104 survivors of arsenic poisoning at the start of the study was 27.4 years (standard deviation, 0.41). The mean length of the follow-up period was 24.3 years, which represented 121 169 person-years (Table 1). During this period, 185 men and 73 women died. We were unable to obtain copies of death certificates from 7 of them, and we classified these 7 cases as deaths due to unknown cause (ICD-9; 799.9). The overall SMR was not significantly higher than that of the general population in Osaka (males: 1.0, 95% CI, 0.9–1.2; females: 1.2, 95% CI, 1.0–1.6; Table 1).

**Table 1.** Number of deaths (*n*) and SMRs for major causes of death among the subjects

	Men		Women		Total	
			
No. of subjects	3738		2366		6104	
Mean age at start of study (yr) (SD)	27.4 (0.4)		27.4 (0.4)		27.4 (0.4)	
Mean duration of follow-up (yr) (SD)	24.3 (2.4)		24.4 (2.0)		24.3 (2.2)	

Cause of death	*n*	SMR (95% CI)	*n*	SMR (95% CI)	*n*	SMR (95% CI)
			
All causes	185	1.0 (0.9–1.2)	73	1.2 (1.0–1.6)	258	1.1 (1.0–1.2)
Infection	0	0.0 (—)	0	0.0 (—)	0	0.0 (—)
All cancers	49	1.0 (0.7–1.3)	30	1.0 (0.7–1.5)	79	1.0 (0.8–1.2)
Stomach	12	1.2 (0.6–2.0)	4	0.7 (0.2–1.6)	16	1.0 (0.6–1.6)
Lung	11	1.3 (0.6–2.2)	2	0.8 (0.1–2.3)	13	1.2 (0.6–1.9)
Endocrine or metabolic disease	3	0.9 (0.2–2.3)	2	2.4 (0.2–7.2)	5	1.2 (0.4–2.6)
Psychiatric disease	1	1.2 (0.0–4.9)	0	0.0 (—)	1	1.0 (0.0–3.9)
Disease of the nervous system	8	3.4^b^ (1.5–6.3)	4	4.5^a^ (1.2–9.9)	12	3.7^b^ (1.9–6.2)
Circulatory disease	40	1.1 (0.8–1.4)	12	1.3 (0.7–2.2)	52	1.1 (0.8–1.5)
Cerebrovascular disease	11	0.9 (0.5–1.6)	4	1.1 (0.3–2.3)	15	1.0 (0.5–1.5)
Respiratory disease	9	1.3 (0.6–2.3)	4	1.8 (0.5–4.0)	13	1.4 (0.8–2.3)
Disease of digestive organs	10	0.7 (0.3–1.1)	1	0.5 (0.0–1.8)	11	0.6 (0.3–1.1)
Skeletal muscle/connective tissue disease	1	2.1 (0.0–7.8)	2	3.0 (0.3–8.2)	3	2.6 (0.5–6.7)
Congenital anomalies	1	2.4 (0.0–9.3)	0	0.0 (—)	1	1.6 (0.0–6.5)
External causes	52	1.0 (0.7–1.3)	14	1.4 (0.8–2.2)	66	1.1 (0.8–1.3)
Traffic accident	11	1.3 (0.6–2.2)	4	3.5 (0.9–8.1)	15	1.6 (0.9–2.5)
Suicide	26	0.9 (0.6–1.2)	8	1.2 (0.5–2.3)	34	0.9 (0.6–1.3)

^a^*P* < 0.05, ^b^*P* < 0.01.

SMR: standardized mortality ratio, SD: standard deviation.

The leading cause of death among the subjects was malignant neoplasm (*n* = 79), although the risk for this cause of death was not significantly elevated (Table 1). The most prevalent cancer site was the stomach (*n* = 16), followed by the lung (*n* = 13) and liver (*n* = 10), although the risks for cancers at these sites were not elevated. No subject suffered death by kidney cancer or skin cancer. The second leading cause of death was death due to external causes (*n* = 66), which was not associated with excess mortality (SMR: 1.1, 95% CI, 0.8–1.3). Of the deaths due to external causes, a marginally elevated mortality risk due to traffic accident was observed (1.6, 0.9–2.5). The third leading cause of death was circulatory diseases (*n* = 52), which was not associated with a significantly increased risk of death (1.1, 0.8–1.5). These deaths were due to acute heart failure without any additional information on the cause of death (*n* = 14), heart failure without any additional information on the cause of death (*n* = 12), acute myocardial infarction (*n* = 7), hypertensive heart disease (*n* = 1), pericarditis (*n* = 1), dilated cardiomyopathy (*n* = 1), arrthymia (*n* = 1), cerebral hemorrhage (*n* = 7), subarachnoid hemorrhage (*n* = 4), cerebral infarction (*n* = 3), and pulmonary infarction (*n* = 1).

We observed significantly increased mortality from diseases of the nervous system (SMR: 3.7, 95% CI, 1.9–6.2, *n* = 12). The most prevalent cause was asphyxia due to epileptic attack (*n* = 4), followed by respiratory and circulatory disorders due to cerebral palsy (*n* = 3), progressive muscular dystrophy (*n* = 2), intracranial hemorrhage due to akinetic epilepsy (*n* = 1), heart failure due to cerebral palsy (*n* = 1), and amyotrophic lateral sclerosis (*n* = 1).

When the subjects were categorized according to duration of follow-up, there was significant excess overall mortality within 5 years from study enrollment (SMR: 1.7, 95% CI, 1.2–2.4), and between 6 and 10 years after study enrollment (1.5, 1.1–2.1; Table 2). Overall mortality then decreased to unity beyond 10 years after study enrollment.

**Table 2.** Number of deaths (*n*) and SMRs among the subjects, by years elapsed since study entry, in 1982

Cause of death	0–4 yrs		5–9 yrs		10–14 yrs		>15 yrs	
			
	*n*	SMR (95% CI)	*n*	SMR (95% CI)	*n*	SMR (95% CI)	*n*	SMR (95% CI)
Men	24	1.6^a^ (1.0–2.3)	30	1.6^a^ (1.1–2.2)	27	0.9 (0.6–1.3)	104	0.9 (0.7–1.1)
Women	12	2.2^a^ (1.1–3.5)	10	1.4 (0.7–2.4)	11	1.1 (0.6–1.9)	40	1.1 (0.8–1.5)
Total	36	1.7^b^ (1.2–2.4)	40	1.5^a^ (1.1–2.1)	38	1.0 (0.7–1.3)	144	0.9 (0.8–1.1)

^a^*P* < 0.05, ^b^*P* < 0.01.

SMR: standardized mortality ratio.

Table 3 presents the risk of death according to employment status at the time of enrollment. Unemployed men had significantly higher overall mortality (2.8, 2.1–3.6) than unity, whereas there was a significantly lower overall mortality among men who were employed (0.8, 0.6–0.9). Unemployed men also had significantly higher mortality from diseases of the nervous system (25.3, 10.8–58.8), respiratory diseases (8.6, 3.1–16.8), circulatory diseases (3.2, 1.6–5.2) and deaths from external causes (2.6, 1.4–4.1). There was no significant excess mortality among women who were employed at the time of enrollment, except for an increased risk of death from circulatory diseases (2.2, 1.3–6.0). Unemployed women had no significant excess all-cause mortality (1.2, 0.9–1.5), but had significant excess mortality from diseases of the nervous system (6.2, 1.7–14.8).

**Table 3.** Number of deaths (*n*) and SMRs from major causes of death among the subjects, by employment status

	Men				Women			
		
	Employed		Unemployed		Employed		Unemployed	
				
No. of subjects	3301		408		587		1742	
Mean age at start of study (yr) (SD)	27.4 (0.4)		27.4 (0.5)		27.4 (0.4)		27.4 (0.4)	
Mean duration of follow-up (yr) (SD)	24.5 (1.7)		23.4 (3.5)		24.5 (1.6)		24.4 (2.0)	

Cause of death	*n*	SMR (95% CI)	*n*	SMR (95% CI)	*n*	SMR (95% CI)	*n*	SMR (95% CI)
				
All causes	119	0.8^b^ (0.6–0.9)	51	2.8^b^ (2.1–3.6)	19	1.3 (0.8–2.0)	50	1.2 (0.9–1.5)
Infection	0	0.0 (—)	0	0.0 (—)	0	0.0 (—)	0	0.0 (—)
All cancers	43	1.0 (0.7–1.3)	4	0.8 (0.2–1.7)	7	1.0 (0.4–1.9)	21	1.0 (0.6–1.5)
Endocrine or metabolic disease	1	0.4 (0.0–1.4)	2	6.1 (0.6–19.1)	1	4.9 (0.0–19.6)	1	1.7 (0.0–6.5)
Psychiatric disease	0	0.0 (—)	0	0.0 (—)	0	0.0 (—)	0	0.0 (—)
Disease of the nervous system	2	1.0 (0.1–2.9)	6	25.3^b^ (10.8–58.8)	0	0.0 (—)	4	6.2^a^ (1.7–14.8)
Circulatory disease	26	0.8 (0.5–1.1)	12	3.2^b^ (1.6–5.2)	7	2.2^a^ (1.3–6.0)	5	0.8 (0.2–1.6)
Cerebrovascular disease	10	1.0 (0.5–1.7)	1	0.8 (0.0–3.3)	3	3.3 (0.6–8.2)	1	0.4 (0.0–1.5)
Respiratory disease	2	0.3 (0.0–1.0)	6	8.6^b^ (3.1–16.8)	1	1.8 (0.0–7.8)	3	1.9 (0.4–4.6)
Disease of digestive organs	7	0.5 (0.2–1.0)	2	1.3 (0.1–3.8)	1	1.9 (0.0–7.8)	0	0.0 (—)
Skeletal muscle/Connective tissue disease	1	2.4 (0.0–9.8)	0	0.0 (—)	0	0.0 (—)	1	2.1 (0.0–7.8)
Congenital anomalies	0	0.0 (—)	1	23.0 (0.0–98.0)	0	0.0 (—)	0	0.0 (—)
External causes	31	0.7^a^ (0.5–0.9)	14	2.6^b^ (1.4–4.1)	2	0.8 (0.1–2.3)	11	1.5 (0.7–2.5)
Traffic accident	8	1.1 (0.5–2.0)	1	1.1 (0.0–4.4)	2	7.2 (0.6–19.1)	2	2.4 (0.2–7.2)
Suicide	17	0.6 (0.4–1.0)	6	2.0 (0.7–3.8)	0	0.0 (—)	7	1.5 (0.6–2.9)

The subjects were classified into 2 groups according to whether they were employed at the time of enrollment, in 1982–1984.

^a^*P* < 0.05, ^b^*P* < 0.01.

SMR: standardized mortality ratio, SD: standard deviation.

Appendix Tables A1–A3 show the SMRs of the 5007 study subjects who were actively followed-up by the Foundation until the end of 2006. The SMR from all causes of death was 1.3 (95% CI, 1.1–1.5). Significant excess mortality in both sexes was observed from diseases of the nervous system (4.7, 2.4–7.6), traffic accident (1.9, 1.1–3.1), and circulatory diseases (1.3, 1.0–1.8). Excess mortality from all causes of death decreased to unity beyond 10 years after study enrollment. The 349 males who were unemployed at the time of enrollment had significantly elevated risks of death from diseases of the nervous system (29.9, 10.8–58.8), respiratory diseases (10.2, 3.6–19.6), circulatory diseases (3.7, 1.9–6.2), and external causes (2.9, 1.5–4.7).

## DISCUSSION

This prospective cohort study showed that, as compared with the general population, individuals who had ingested arsenic-contaminated Morinaga Dry Milk when they were younger than 2 years and had survived for 27 years still had increased overall mortality for an additional 10 years; however, the excess overall mortality decreased to unity when they reached middle age. The current study also demonstrated that subjects had significantly higher mortality from diseases of the nervous system than the general population, and that overall mortality among men who were unemployed at enrollment was 2.8 times that of the general population. Previous studies of poisoning from ingested arsenic described 15 fatalities among 500 patients who had ingested arsenic-contaminated wine in France in 1888, 70 fatalities among 6000 persons who had ingested arsenic-contaminated beer in England in 1900–1901, and 15 fatalities among 28 patients who had ingested arsenic-contaminated cider in the United States in 1924.^19^ These fatalities were cases of acute poisoning.

Epidemiological studies of long-term ingestion of arsenic-contaminated water have been conducted in Taiwan,^2^^–^^4^^,^^7^^,^^12^ Chile,^8^ Argentina,^5^^,^^6^ Japan,^9^ the United Kingdom,^10^ and the United States.^11^ These studies found elevated risks of skin, lung, bladder, kidney, and liver cancers. Our results did not reveal an increased risk of cancer death at any specific site, which might be due to differences in the duration of exposure, observed age at risk, or the survivor effect. However, because the age of the exposed subjects was only approximately 50 years, the expected number of cancer cases was low, and a statistically significant increase would therefore be difficult to detect. To evaluate excess cancer risk in the present subjects, we need to continue follow-up for at least another 10 years.

The elevated mortality from diseases of the nervous system was a notable finding in the current study. This elevated mortality risk was mainly caused by epilepsy and cerebral palsy. Nishida et al^23^ performed a physical examination of 47 victims of the Morinaga Dry Milk arsenic poisoning who had any symptom in 1970 (when they were approximately 15 years of age) and reported that 11 had symptoms of central nervous system disorders. Their report, together with our results, indicates that acute arsenic poisoning through ingestion of arsenic-contaminated dry milk in infancy can permanently damage the central nervous system. The extremely high mortality rate from diseases of the nervous system among men who were unemployed at the time of enrollment suggests that damage to the central nervous system due to acute arsenic poisoning made it difficult for these men to find or maintain employment in their 20s. However, we cannot exclude the possibility of contamination by another toxic agent that might have induced nervous system disorders in the victims.

Mortality from traffic accidents among the study subjects was 1.6 times that of the general population, although the difference was statistically insignificant. Ohira and Aoyama^24^ conducted a community-based survey in Hiroshima in 1971 and found that, as compared with control children, victims of arsenic poisoning from Morinaga Dry Milk had a higher prevalence of narrowing of the visual field caused by macular degeneration. A recent cross-sectional study in Taiwan reported that chronic exposure to arsenic in drinking water was related to the occurrence of pterygium, which can potentially lead to blindness.^24^ In addition, motor or sensory dysfunction due to the aftereffects of arsenic poisoning might result in a traffic accident. To prevent accidents, we need to assess the motor, sensory, and optical function of victims.

There were several methodological limitations in the current study. First, since there were no quantitative data on individual levels of arsenic ingestion, we could not analyze the dose-response relationship between the level of exposure and risk of death. Second, we included 1097 individuals who declined further active contact by the Foundation before the end of 2006. We assume that many of them declined further contact at the time of employment or marriage, to avoid being notified of their accident history while they were developing new relationships. Therefore, such persons were probably healthier than subjects who continued to be actively followed by the Foundation. In addition, since those subjects whose families did not request condolence money during the follow-up were assumed to be alive until the closing date, their inclusion might have led to an underestimation of current excess mortality. The results of our additional analyses that exclude the 1097 individuals (Appendix) indicate that there was underestimation of current excess mortality risk. Third, attrition bias and the survivor effect in our cohort study might have led to underestimation of current SMRs. Fourth, the risk assessment for mortality might be affected by potential confounding factors that were not investigated in our study (ie, environmental risk factors and socioeconomic factors like parental income). Fifth, there were 7 subjects who died during the study period for whom copies of death certificates were not obtained. This might have led to underestimation of some cause-specific SMRs, although the effect would be limited. Last, given the rather large number of analyses, some significant findings might have been due the chance effect of multiple comparisons.

In conclusion, this long-term prospective cohort study revealed overall and cause-specific mortality risks among survivors who ingested arsenic-contaminated dry milk during infancy. The results indicate that food safety considerations are very important, particularly with regard to large-scale production of foods for infants. Further prospective follow-up studies of these subjects are necessary to assess risks when the victims are in their 50s and to establish appropriate health programs for their aid.

## APPENDIX

Tables A1 to A3 show the SMRs of the 5007 study subjects who continued to be actively followed by the Foundation until the end of 2006.

**Table A1.** Number of deaths (*n*) and SMRs from major causes of death among the subjects

	Men		Women		Total	
			
No. of subjects	3085		1922		5007	
Mean age at start of study (yr) (SD)	27.4 (0.4)		27.4 (0.4)		27.4 (0.4)	
Mean duration of follow-up (yr) (SD)	24.2 (2.3)		24.4 (1.9)		24.2 (2.2)	

Cause of death	*n*	SMR (95% CI)	*n*	SMR (95% CI)	*n*	SMR (95% CI)
			
All causes	177	1.2^a^ (1.0–1.4)	70	1.5^b^ (1.2–1.9)	247	1.3^b^ (1.1–1.5)
Infection	0	0.0 (—)	0	0.0 (—)	0	0.0 (—)
All cancers	47	1.2 (0.9–1.5)	30	1.3 (0.9–1.8)	77	1.2 (1.0–1.5)
Stomach	10	1.2 (0.6–2.1)	4	0.9 (0.2–2.1)	14	1.1 (0.6–1.8)
Lung	11	1.6 (0.8–2.7)	2	1.0 (0.1–2.9)	13	1.5 (0.8–2.4)
Endocrine or metabolic disease	3	1.2 (0.2–2.8)	2	3.0 (0.3–8.2)	5	1.5 (0.5–3.2)
Psychiatric disease	1	1.5 (0.0–5.6)	0	0.0 (—)	1	1.3 (0.0–4.9)
Disease of the nervous system	8	4.3^b^ (3.8–16.1)	4	5.7^a^ (1.5–12.7)	12	4.7^b^ (2.4–7.6)
Circulatory disease	39	1.3 (0.9–1.7)	11	1.5 (0.8–2.6)	50	1.3^a^ (1.0–1.7)
Cerebrovascular disease	10	1.1 (0.5–1.8)	3	1.0 (0.2–2.5)	13	1.0 (0.6–1.7)
Respiratory disease	9	1.6 (0.7–2.9)	3	1.7 (0.3–4.1)	12	1.7 (0.9–2.7)
Disease of digestive organs	7	0.6 (0.2–1.1)	1	0.6 (0.0–2.3)	8	0.6 (0.2–1.1)
Skeletal muscle/connective tissue disease	1	2.6 (0.0–9.8)	2	3.7 (0.4–11.5)	3	3.3 (0.6–8.2)
Congenital anomalies	1	3.0 (0.0–13.1)	0	0.0 (—)	1	2.0 (0.0–7.8)
External causes	50	1.2 (0.9–1.5)	13	1.6 (0.9–2.6)	63	1.3 (1.0–1.6)
Traffic accident	11	1.6 (0.8–2.7)	4	4.4^a^ (1.2–9.9)	15	1.9^a^ (1.1–3.1)
Suicide	24	1.0 (0.6–1.4)	7	1.4 (0.5–2.6)	31	1.1 (0.7–1.5)

^a^*P* < 0.05, ^b^*P* < 0.01.

SMR: standardized mortality ratio, SD: standard deviation.

**Table A2.** Number of deaths (*n*) and SMRs from major causes of death among the subjects, by years elapsed since study entry, in 1982

Cause of death	0–4 yrs		5–9 yrs		10–14 yrs		>15 yrs	
			
	*n*	SMR (95% CI)	*n*	SMR (95% CI)	*n*	SMR (95% CI)	*n*	SMR (95% CI)
All causes	36	2.2^b^ (1.5–2.9)	39	1.9^b^ (1.3–2.5)	37	1.2 (0.8–1.6)	135	1.1 (0.9–1.3)
All cancers	5	1.6 (0.5–3.3)	5	0.9 (0.3–1.9)	12	1.2 (0.6–2.0)	55	1.2 (0.9–1.6)
Stomach	2	2.1 (0.2–5.7)	1	0.7 (0.0–2.6)	2	0.9 (0.1–2.6)	9	1.2 (0.5–2.1)
Lung	0	0.0 (—)	1	2.1 (0.0–8.0)	4	3.0 (0.8–6.8)	8	1.2 (0.5–2.1)
Disease of the nervous system	3	9.1^a^ (1.9–24.5)	2	5.9 (0.6–19.1)	3	7.6^a^ (1.4–18.4)	4	2.6 (0.7–5.9)
Circulatory disease	6	2.4 (0.9–4.7)	13	3.5^b^ (1.8–5.5)	6	1.0 (0.4–1.9)	25	1.0 (0.7–1.4)
Cerebrovascular disease	2	3.4 (0.3–9.6)	2	1.8 (0.2–5.2)	0	0.0 (—)	9	1.0 (0.5–1.8)
Respiratory disease	1	1.4 (0.0–5.6)	1	1.2 (0.0–4.9)	0	0.0 (—)	10	2.2^a^ (1.1–3.8)
External causes	13	1.7 (0.9–2.7)	13	1.7 (0.9–2.8)	13	1.5 (0.8–2.5)	24	0.9 (0.6–1.3)
Traffic accident	5	2.9 (0.9–6.1)	3	1.8 (0.4–4.6)	1	0.6 (0.0–2.5)	6	2.2 (0.8–4.2)
Suicide	7	1.7 (0.7–3.2)	5	1.3 (0.4–2.7)	8	1.7 (0.7–3.2)	11	0.7 (0.3–1.1)

^a^*P* < 0.05, ^b^*P* < 0.01.

SMR: standardized mortality ratio.

**Table A3.** Number of deaths (*n*) and SMRs from major causes of death among the subjects, by employment status

	Men				Women			
		
	Employed		Unemployed		Employed		Unemployed	
				
No. of subjects	2707		349		485		1415	
Mean age at start of study (yr) (SD)	27.4 (0.39)		27.4 (0.49)		27.4 (0.41)		27.4 (0.42)	
Mean duration of follow-up (yr) (SD)	24.4 (1.67)		23.2 (3.52)		24.4 (1.55)		24.4 (1.98)	

Cause of death	*n*	SMR (95% CI)	*n*	SMR (95% CI)	*n*	SMR (95% CI)	*n*	SMR (95% CI)
				
All causes	114	0.9 (0.7–1.1)	50	3.2^b^ (2.4–4.2)	18	1.5 (0.9–2.3)	48	1.4^a^ (1.0–1.8)
Infection	0	0.0 (—)	0	0.0 (—)	0	0.0 (—)	0	0.0 (—)
All cancers	41	1.1 (0.8–1.5)	4	0.9 (0.2–2.1)	7	1.2 (0.5–2.3)	21	1.3 (0.8–1.9)
Endocrine or metabolic disease	1	0.4 (0.0–1.7)	2	7.3 (0.6–19.1)	1	6.0 (0.0–19.6)	1	2.1 (0.0–7.8)
Psychiatric disease	0	0.0 (—)	0	0.0 (—)	0	0.0 (—)	0	0.0 (—)
Disease of the nervous system	2	1.2 (0.1–3.4)	6	29.9^b^ (10.8–58.8)	0	0.0 (—)	4	7.7^a^ (2.1–17.8)
Circulatory disease	25	0.9 (0.6–1.3)	12	3.7^b^ (1.9–6.2)	6	3.3^a^ (1.2–6.5)	5	0.9 (0.3–2.0)
Cerebrovascular disease	9	1.1 (0.5–1.9)	1	1.0 (0.0–3.9)	2	2.6 (0.2–7.2)	1	0.5 (0.0–1.8)
Respiratory disease	2	0.4 (0.0–1.2)	6	10.2^b^ (3.6–19.6)	1	2.2 (0.0–9.8)	2	1.5 (0.1–4.4)
Disease of digestive organs	5	0.5 (0.1–1.0)	2	1.5 (0.1–4.4)	1	2.3 (0.0–9.8)	0	0.0 (—)
Skeletal muscle/Connective tissue disease	1	3.0 (0.0–13.1)	0	0.0 (—)	0	0.0 (—)	1	2.5 (0.0–9.8)
Congenital anomalies	0	0.0 (—)	1	27.1 (0.0–98.0)	0	0.0 (—)	0	0.0 (—)
External causes	31	0.8 (0.6–1.2)	13	2.9^b^ (1.5–4.7)	2	1.0 (0.1–2.9)	10	1.7 (0.8–2.9)
Traffic accident	8	1.3 (0.6–2.4)	1	1.4 (0.0–5.6)	2	8.7^a^ (1.1–33.7)	2	3.0 (0.3–8.2)
Suicide	17	0.8 (0.5–1.2)	5	1.9 (0.6–4.0)	0	0.0 (—)	6	1.6 (0.6–3.2)

The subjects were classified into 2 groups according to whether they were employed at the time of enrollment, in 1982–1984.

^a^*P* < 0.05, ^b^*P* < 0.01.

SMR: standardized mortality ratio, SD: standard deviation.
